# Stress-Induced Enhancement of Ethanol Intake in C57BL/6J Mice with a History of Chronic Ethanol Exposure: Involvement of Kappa Opioid Receptors

**DOI:** 10.3389/fncel.2016.00045

**Published:** 2016-02-23

**Authors:** Rachel I. Anderson, Marcelo F. Lopez, Howard C. Becker

**Affiliations:** ^1^Medical University of South CarolinaCharleston, SC, USA; ^2^Charleston Alcohol Research CenterCharleston, SC, USA; ^3^Ralph H. Johnson Veterans Administration Medical CenterCharleston, SC, USA

**Keywords:** ethanol, stress, kappa opioid receptor, dynorphin, mice

## Abstract

Our laboratory has previously demonstrated that daily forced swim stress (FSS) prior to ethanol drinking sessions facilitates enhanced ethanol consumption in mice with a history of chronic intermittent ethanol (CIE) vapor exposure without altering ethanol intake in air-exposed controls. Because both stress and chronic ethanol exposure have been shown to activate the dynorphin/kappa opioid receptor (KOR) system, the present study was designed to explore a potential role for KORs in modulating stress effects on ethanol consumption in the CIE model of dependence and relapse drinking. After stable baseline ethanol intake was established in adult male C57BL/6J mice, subjects received chronic intermittent exposure (16 h/day × 4 days/week) to ethanol vapor (CIE group) or air (CTL group). Weekly cycles of inhalation exposure were alternated with 5-day limited access drinking tests (1 h access to 15% ethanol). Experiment 1 compared effects of daily FSS and KOR activation on ethanol consumption. CIE and CTL mice were either exposed to FSS (10 min), the KOR agonist U50,488 (5 mg/kg), or a vehicle injection (non-stressed condition) prior to each daily drinking session during test weeks. FSS selectively increased drinking in CIE mice. U50,488 mimicked this effect in CIE mice, but also increased drinking in CTL mice. Experiment 2 assessed effects of KOR blockade on stress-induced drinking in CIE and CTL mice. Stressed and non-stressed mice were administered the short-acting KOR antagonist LY2444296 (0 or 5 mg/kg) 30 min prior to each drinking session during test weeks. FSS selectively increased ethanol consumption in CIE mice, an effect that was abolished by LY2444296 pretreatment. In Experiment 3, CIE and CTL mice were administered one of four doses of U50,488 (0, 1.25, 2.5, 5.0 mg/kg) 1 h prior to each daily drinking test (in lieu of FSS). All doses of U50,488 increased ethanol consumption in both CIE and CTL mice. The U50,488-induced increase in drinking was blocked by LY2444296. Our results demonstrate that the KOR system contributes to the stress enhancement of ethanol intake in mice with a history of chronic ethanol exposure.

## Introduction

It is generally recognized that stress exposure may significantly contribute to the initiation of ethanol drinking and perpetuate continued ethanol use (Brady and Sonne, 1999). In this way, stress may facilitate transition from social (regulated) ethanol use to more excessive levels of consumption that can lead to the development of alcohol dependence in humans (Uhart and Wand, 2009). In ethanol-dependent individuals attempting abstinence, stress is known to serve as a potent trigger for relapse drinking (Sinha, 2012). Increased relapse vulnerability continues to be a major impediment to treatment of alcohol use disorders. Elucidation of the underlying mechanisms that contribute to stress-induced drinking may ultimately lead to the development of new and more effective treatment strategies for alcoholism. Although demonstrating stress-induced increases in ethanol consumption using animal models has proven challenging (cf. Becker et al., 2011), our laboratory has recently developed a model that demonstrates stress-induced enhancement of ethanol drinking in mice with a history of chronic intermittent ethanol (CIE) exposure (Lopez et al., 2016). As such, this model is well suited for studying mechanisms that contribute to this apparent unique interaction of stress with ethanol in the context of dependence.

The endogenous opioid system, which includes endorphins, enkephalins, and dynorphins, has been widely implicated in the motivational effects of ethanol through actions at mu, delta, and kappa opioid receptors (KORs). While mu and delta receptors influence ethanol’s rewarding effects, ethanol dependence-related drinking has been shown to be more sensitive to antagonism by KOR antagonists (Kissler et al., 2014). Several reports have indicated that naltrexone, a general opioid antagonist with greatest affinity for mu receptors, does not attenuate stress-induced reinstatement of operant responding for ethanol (Lê et al., 1999; Liu and Weiss, 2002). On the other hand, converging molecular and behavioral evidence suggests that KORs may pose a promising therapeutic target for attenuation of stress-related ethanol consumption in alcohol-dependent subjects.

KORs and their endogenous ligands, dynorphins (derived from the precursor prodynorphin), are sensitive to both chronic ethanol and stress exposure. For example, exposure to various stressors has been shown to increase immunoreactivity of Dynorphin A and Dynorphin B in regions of the hippocampus and nucleus accumbens (Shirayama et al., 2004). Similarly, 24 h after forced swim stress (FSS) exposure, prodynorphin immunoreactivity is increased in the dorsal bed nucleus of the stria terminalis (dBNST) and the lateral central nucleus of the amygdala (CeA; Chung et al., 2014), with elevated prodynorphin mRNA in the nucleus accumbens also reported (Chartoff et al., 2009). Various ethanol exposure regimens that involve both experimenter-administered as well as self-administered ethanol exposure have been reported to increase prodynorphin mRNA levels and Dynorphin B expression in the nucleus accumbens (Przewłocka et al., 1997; Lindholm et al., 2000). More recent work has also shown that KOR mRNA, preprodynorphin mRNA, and dynorphin signaling are upregulated in the amygdala following chronic ethanol exposure (D’Addario et al., 2013; Zhou et al., 2013; Kissler et al., 2014). Thus, there is a convergent body of literature that indicates both stress and ethanol exposure activate the dynorphin/KOR system.

Supporting a role for the dynorphin/KOR system in behaviors related to stress, activation of KORs has been shown to increase behavioral measures of anxiety-like and depressive-like states. For example, the KOR agonist U50,488 reduced open arm time in the elevated plus maze in both mice (Bruchas et al., 2009) and rats (Valdez and Harshberger, 2012). Complementary studies in transgenic mice have reported that genetic deletion of dynorphin produced an anxiolytic profile, as indexed by dynorphin knock-out mice spending more time in the light side during a light-dark box task relative to their wild-type counterparts (Wittmann et al., 2009). KOR agonists have also been shown to increase immobility in the FSS model of depressive-like behavior whereas KOR antagonists typically reduce immobility (Mague et al., 2003; McLaughlin et al., 2003; Beardsley et al., 2005; Zhang et al., 2005; Carlezon et al., 2006; Carey et al., 2009).

Exposure to various stressors has been previously shown to increase the rewarding effects of ethanol and decrease the aversive properties of ethanol (Matsuzawa et al., 1998; Funk et al., 2004; Sperling et al., 2010). For example, exposure to FSS was shown to enhance conditioned place preference induced by ethanol, nicotine, and cocaine (Schindler et al., 2010; Sperling et al., 2010; Smith et al., 2012). Interestingly, activation of KORs was reported to mimic this stress-induced enhancement of conditioned drug-related reward states. That is, systemic administration of the KOR agonist U50,488 resulted in a potentiation of ethanol-induced conditioned place preference similar to that produced by FSS in mice (Sperling et al., 2010). Comparable effects were observed with cocaine and nicotine (McLaughlin et al., 2006; Schindler et al., 2010; Smith et al., 2012). Additionally, pharmacological activation of KORs was shown to reinstate ethanol responding in an operant conditioning model of relapse (Funk et al., 2014). Cues associated with central administration of U50,488 have also been shown to increase ethanol self-administration in nondependent rats (Berger et al., 2013).

In another series of studies, blockade of KORs was shown to prevent escalation of ethanol self-administration induced by CIE vapor exposure in rats. Specifically, the long-acting KOR antagonist nor-binaltorphimine (nor-BNI) reduced ethanol self-administration in rats exposed to CIE vapor, but not air-exposed controls after either systemic or central administration (Walker and Koob, 2008; Walker et al., 2011). Further, Walker and colleagues have implicated the CeA as a locus of KOR-mediated effects by demonstrating that site-specific infusion of nor-BNI into the CeA reduces ethanol consumption in chronic ethanol-exposed rats (Kissler et al., 2014).

Given that the dynorphin/KOR system plays a role in mediating chronic ethanol effects as well as behavioral effects of stress, the current studies were designed to investigate the role of KORs in stress-enhanced drinking in subjects with a history of CIE exposure. Specifically, the effects of KOR activation were compared with the effects of daily FSS exposure on voluntary ethanol consumption in mice with a history of CIE vapor exposure and air-exposed control (CTL) mice. FSS was selected based on our previous findings that this stressor is more effective at increasing ethanol consumption in the CIE drinking model (Lopez et al., 2016). It was hypothesized that systemic administration of a KOR agonist would mimic the effects of FSS to augment drinking in CIE-exposed mice. Additionally, effects of KOR blockade on FSS-enhanced drinking were also evaluated in CIE and CTL mice.

## Materials and Methods

### Subjects

A total of 231 male C57BL/6J mice ordered from Jackson Laboratories (Bar Harbor, ME, USA) were individually housed in standard polycarbonate cages with corncob bedding in a temperature- and humidity-controlled AAALAC-accredited facility. Subjects were maintained on a 12 h modified reverse light/dark cycle with ad libitum access to food and water throughout experimentation. Mice were at least 10 weeks old at the start of each experiment. Subjects were treated in accordance with the NIH Guide for the Care and Use of Laboratory Animals (8th edition, National Research Council, 2011) under protocols approved by the Institutional Animal Care and Use Committee at the Medical University of South Carolina.

### Drugs

The KOR agonist U50,488 (Tocris) was dissolved in saline and briefly sonicated. Doses of 1.25–5.0 mg/kg were administered intraperitoneally (i.p.) in a volume of 10 ml/kg body weight. The novel, short-acting KOR antagonist LY2444296 ([(S)-3-fluoro-4-(4-((2-(3-fluorophenyl)pyrrolidin-1-yl)methyl) phenoxy)benzamide]; compound #25 described in Mitch et al., 2011) was synthesized at Eli Lilly Research Laboratories (Indianapolis, IN, USA) and was dissolved in a saline/polysorbate 80 mixture and sonicated. LY2444296 (5 mg/kg) was also delivered via i.p. injection (10 ml/kg body weight).

### Chronic Intermittent Ethanol (CIE) Exposure and Drinking Procedure

Starting 3 h into the dark cycle, subjects were given access to one bottle containing 15% ethanol (v/v) for 1 h. After 4–6 weeks of baseline drinking, subjects were matched for average ethanol consumption during the final week of baseline and assigned to an ethanol exposure condition. Mice were then subjected to repeated weekly cycles of CIE vapor or air (CTL) exposure, with 5-day ethanol drinking test sessions conducted during intervening weeks. Each weekly cycle of CIE (or air) inhalation exposure occurred in Plexiglas chambers and consisted of 16 h/day exposure for 4 consecutive days followed by a 3-day forced abstinence period before the start of each drinking test week, as previously described (Griffin et al., 2009).

**Figure 1:**
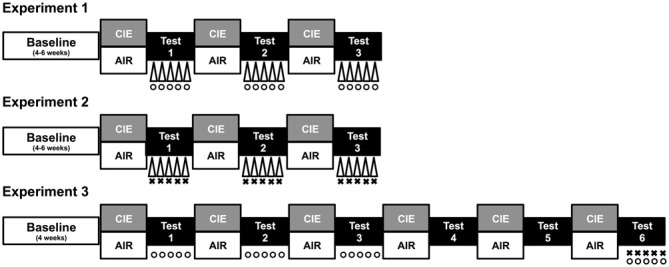
**Procedure overview.** Each study consisted of a period of baseline ethanol consumption followed by repeated 1-week cycles of CIE (or air) exposure alternated with 1-week drinking test weeks. During test weeks in Experiment 1, CIE and CTL mice were subjected to one of three conditions: a 10-min forced swim stress (FSS) exposure 4 h before drinking (indicated by Δ), an injection of the KOR agonist U50,488 (5 mg/kg) 1 h before drinking (indicated by °), or a saline injection 1 h before drinking (non-stressed control group). During each test week in Experiment 2, CIE and CTL mice were assigned to one of four conditions defined by the 2 × 2 design: FSS or no stress (indicated by Δ) 4 h before drinking and an injection of LY2444296 (5 mg/kg) or vehicle 30 min before drinking (indicated by ×). During Tests 1–3 of Experiment 3, CIE and CTL mice received an injection of U50,488 (0, 1.25, 2.5, or 5 mg/kg) 1 h before drinking (indicated by °). Mice were not treated during Tests 4–5 (washout). During Test 6, CIE and CTL mice were assigned to one of four conditions: pretreatment with LY2444296 (5 mg/kg) or vehicle 1.5 h before drinking (indicated by ×) and administration of U50,488 (5 mg/kg) or vehicle 1 h before drinking (indicated by °).

Prior to placement in ethanol vapor chambers for each 16 h exposure, subjects in the CIE condition received a loading dose of ethanol (1.6 g/kg) mixed with the alcohol dehydrogenase inhibitor pyrazole (1 mmol/kg) via intraperitoneal injection (20 ml/kg). Ethanol was administered to initiate intoxication and pyrazole was given to slow the clearance of ethanol, thereby maintaining high, stable blood ethanol levels throughout each of the 16 h bouts of ethanol vapor exposure (Griffin et al., 2009). Chamber ethanol concentrations were monitored daily and blood samples were collected from the retroorbital sinus on a weekly basis to ensure that ethanol exposure resulted in blood ethanol concentrations (BECs) within the target range of 175–225 mg/dl. All blood samples were centrifuged and the plasma was assayed using an Analox ethanol analyzer. Mice in the CTL condition were handled similarly, but received injections of pyrazole mixed in saline (rather than ethanol) prior to air exposure in inhalation chambers. A procedure overview for each study is depicted in Figure 1 and described in detail below.

### Forced Swim Stress Procedure

Mice assigned to the stress condition were exposed to forced swim stress (FSS) for 10 min each day during test weeks following inhalation exposure. FSS exposure occurred in glass cylinders (40 cm high, 20 cm diameter) that were half-filled with 23–25°C tap water and separated by foam board dividers. Upon removal from the cylinders, subjects were hand-dried and allowed to recover on a heating pad for 5–10 min before returning to the colony room. Water in each cylinder was replaced between subjects.

### Experiment 1

This experiment was designed to directly compare the effects of daily FSS exposure and daily administration of the KOR agonist U50,488 on ethanol consumption in CIE and CTL mice. A total of 67 mice were assigned to one of six experimental conditions (*n* = 11–12/group) defined by the 2 Group (CIE, CTL) × 3 Stress (No-Stress, FSS, U50,488) factorial design. FSS exposure occurred 4 h before each daily drinking test session. U50,488 (5 mg/kg, i.p.) was injected 60 min prior to the start of each daily drinking test session. Mice in the no-stress condition received a saline injection at this time.

### Experiment 2

This experiment was designed to examine effects of KOR blockade on ethanol consumption in stressed (FSS) and non-stressed mice with a history of either CIE or air exposure. A total of 84 mice were assigned to one of eight experimental conditions defined by the 2 Group (CIE, CTL) × 2 Stress (No-Stress, FSS) × 2 Dose (0, 5 mg/kg LY2444296) factorial design (*n* = 7–12/group). As in Experiment 1, mice in the stress condition were exposed to 10-min FSS 4 h before each daily test drinking session. Mice also received injections of the KOR antagonist LY2444296 (5 mg/kg, i.p.) or vehicle 30 min prior to the start of the 1 h drinking test sessions. Immediately following the final 1 h drinking session, blood samples were collected and assayed for blood ethanol concentration, as described above.

### Experiment 3

This experiment was designed to generate a dose-response function for the KOR agonist U50,488 to more fully examine the effects of this treatment on ethanol consumption in CIE and CTL groups of mice. A total of 80 mice were used, with 10 mice per experimental condition. During each drinking test week, separate groups of CIE and CTL mice were administered one of four doses of U50,488 (0, 1.25, 2.5, or 5.0 mg/kg, i.p.) 60 min prior to each daily drinking test (*n* = 10/group). After Test Week 3, mice continued with two additional cycles of CIE (or air) exposure, but only saline injections were administered to all mice during Test Weeks 4 and 5 (drug-free washout). Prior to Test Week 6, all subjects within CIE and CTL conditions were re-sorted to equate ethanol intake during Test Week 5 and assigned to new drug treatment conditions based on a 2 LY2444296 Pretreatment (0, 5 mg/kg) × 2 U50,488 Treatment (0, 5 mg/kg) design. LY2444296 pretreatment was given 60 min prior to the 1 h drinking session and 30 min prior to U50,488 treatment.

## Results

### Experiment 1

Drinking data were expressed as average weekly ethanol intake (g/kg) and analyzed by a 3-way ANOVA, with Group (CIE, CTL) and Stress (No-Stress, FSS, U50,488) as between-subject factors and Week (Baseline, Test 1, Test 2, Test 3) as a repeated measure. This analysis revealed main effects of Group [*F*_(1,61)_ = 7.61, *p* < 0.01], Stress [*F*_(2,61)_ = 24.593, *p* < 0.001], and Week [*F*_(3,183)_ = 24.489, *p* < 0.001], in additional to a significant Stress × Week interaction [*F*_(6,183)_ = 8.804, *p* < 0.01]. *Post hoc* analyses indicated that U50,488 significantly increased ethanol consumption relative to baseline levels of intake as well as intake in both No-Stress and FSS groups during all three test weeks (*p*’s < 0.05).

To further explore potential differences between CIE and CTL mice, data were separately analyzed for each ethanol exposure condition. Analysis of CTL mice revealed significant main effects of Stress [*F*_(2,31)_ = 13.784, *p* < 0.001] and Week [*F*_(3,93)_ = 9.945 *p* < 0.001], and a Stress × Week interaction [*F*_(6,93)_ = 5.73, *p* < 0.001]. *Post hoc* tests indicated that U50,488 treatment increased drinking relative to baseline and relative to both No-Stress and FSS groups during all three test weeks. No changes were observed for either FSS-exposed or No-Stress mice.

**Figure 2:**
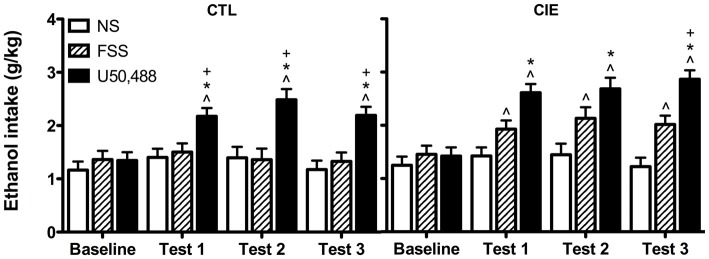
**Average weekly ethanol consumption (g/kg).** Among air-exposed control (CTL) mice, administration of the KOR agonist U50,488 (5 mg/kg) resulted in elevated ethanol intake relative to baseline (indicated by ^∧^), non-stressed (NS) mice (indicated by *), and forced swim stress-exposed (FSS) mice (indicated by ^+^) during all three test weeks. Among mice exposed to chronic intermittent ethanol (CIE), administration of U50,488 resulted in elevated ethanol intake relative to baseline (indicated by ^∧^) and non-stressed mice (indicated by *) during Tests 1–3, and relative to FSS mice during Test 3 (indicated by ^+^). FSS mice demonstrated elevated ethanol consumption relative to baseline during all three test weeks (indicated by ^∧^).

Analysis of CIE mice revealed significant main effects of Stress [*F*_(2,30)_ = 12.428, *p* < 0.001] and Week [*F*_(3,90)_ = 15.154, *p* < 0.001], as well as a Stress × Week interaction [*F*_(6,90)_ = 4.577, *p* < 0.001]. *Post hoc* tests indicated that mice exposed to FSS increased consumption relative to baseline during Tests 1–3. Mice in the U50,488 group increased consumption relative to baseline and relative to the No-Stress group during Tests 1–3 (*p*’s < 0.05). During Test 3, mice in the U50,488 group also consumed more ethanol than mice in the FSS condition (*p* = 0.05). No changes were observed in the No-Stress condition. Data are shown in Figure 2.

### Experiment 2

**Figure 3:**
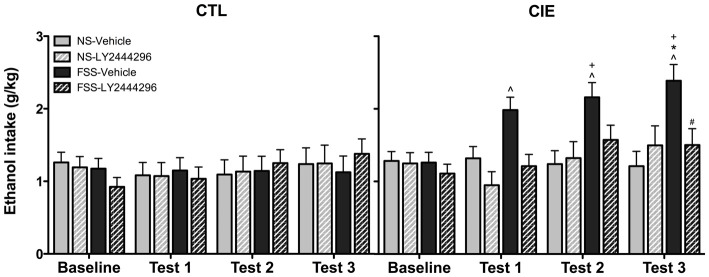
**Average weekly ethanol consumption (g/kg).** Among air-exposed control (CTL) mice, no effects of stress, chronic ethanol exposure (CIE), or KOR blockade were observed. Among CIE-exposed mice, FSS exposure (solid black bars) increased ethanol consumption relative to baseline consumption during all three test weeks (indicated by ^∧^), relative to CTL-FSS mice during Tests 2–3 (indicated by ^+^), and relative to CIE-NS mice during Test 3 (indicated by *). Pretreatment with the KOR antagonist LY2444296 (striped black bars) blocked the FSS-induced increased drinking in CIE mice and resulted in a significant reduction from CIE-FSS vehicle mice during Test 3 (indicated by ^#^).

Drinking data were expressed as average weekly ethanol intake (g/kg) and analyzed using a 2 (Group) × 2 (Stress) × 2 (LY2444296 Dose) × 4 (Week) repeated measures ANOVA. The analysis revealed a significant Group × Stress × Dose × Week interaction [*F*_(3,210)_ = 2.75, *p* < 0.05]. *Post hoc* tests indicated no significant differences in drinking among CTL groups of mice during baseline or any of the test weeks. In contrast, vehicle-treated FSS-exposed mice with a history of CIE exposure demonstrated a significant increase in drinking above baseline levels during all three test weeks. These vehicle-treated CIE-FSS mice also consumed more ethanol than CIE-NS mice during Test 3 and more ethanol than CTL-FSS mice during Tests 2–3 (*p*’s < 0.05). While the KOR antagonist did not alter drinking in CTL mice, pretreatment with LY2444296 abolished FSS-enhanced drinking in CIE-exposed mice during all three test weeks of the study (Figure 3).

**Figure 4:**
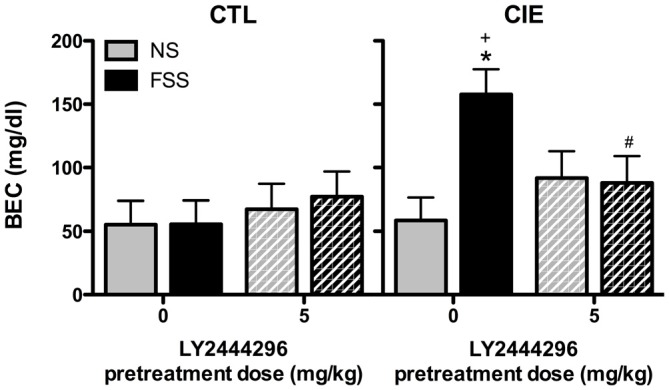
**Blood ethanol concentrations (BECs) following the final drinking session.** Vehicle-treated CIE-FSS mice had higher BECs than CIE-NS mice (indicated by *) and CTL-FSS mice (indicated by ^+^). The FSS-induced increase in BEC was blocked by pretreatment with LY2444296, which resulted in significantly lower BECs than vehicle-treated CIE-FSS mice (indicated by ^#^).

BEC data from the final drinking session during Test 3 were analyzed by a 2 (Group) × 2 (Stress) × 2 (LY2444296 Dose) factorial ANOVA, which revealed a significant Group × Stress × Dose interaction [*F*_(1,58)_ = 4.08, *p* < 0.05]. Further analyses indicated that CIE-FSS mice registered significantly higher BECs than mice in all other conditions (Figure 4). No other group differences were observed.

### Experiment 3

**Figure 5:**
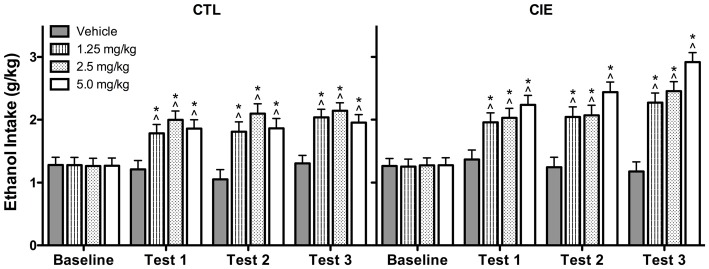
**Average weekly ethanol consumption (g/kg).** All doses of the KOR agonist U50,488 resulted in elevated ethanol consumption relative to baseline drinking (indicated by ^∧^) and vehicle-injected controls (indicated by *) during Tests 1–3 for both CIE and CTL mice.

Average weekly ethanol intake (g/kg) was analyzed using a 2 (Group) × 4 (U50,488 Dose) × 4 (Week) repeated measures ANOVA, which revealed significant main effects of Group [*F*_(1,72)_ = 4.50, *p* < 0.05], Dose [*F*_(3,72)_ = 14.75, *p* < 0.01], and Week [*F*_(3,216)_ = 53.81, *p* < 0.01], as well as Group × Week [*F*_(3,216)_ = 2.82, *p* < 0.05] and Dose × Week [*F*_(9,216)_ = 8.57, *p* < 0.01] interactions. *Post hoc* analyses indicated that U50,488 administration significantly increased ethanol consumption above baseline levels in both CIE and CTL groups during all test weeks. Additionally, U50,488 increased ethanol intake compared to the vehicle condition in CIE and CTL mice, an effect that did not significantly differ as a function of dose. The increase in ethanol consumption induced by treatment with the KOR agonist appeared to be greater in CIE mice compared to CTL mice (especially during the last test week), but this effect did not achieve statistical significance (Figure 5).

Analysis of average weekly ethanol intake (g/kg) during the drug washout evaluation period (Test Weeks 4 and 5; saline injections only) indicated that the effects of U50,488 treatment dissipated prior to the final Test Week. Specifically, ANOVA revealed a significant Dose × Week interaction [*F*_(3,71)_ = 3.361, *p* < 0.05]. *Post hoc* analysis indicated that mice previously exposed to any of the U50,488 doses during Test Weeks 1–3 continued to demonstrate elevated ethanol consumption relative to vehicle-exposed mice during Test Week 4. This residual drug effect was no longer observed the following week (Test Week 5). Thus, the increased ethanol consumption induced by previous U50,488 administration persisted for 1 week beyond treatment (Test 4) before subsiding during the second washout week (Test 5; data not shown).

**Figure 6:**
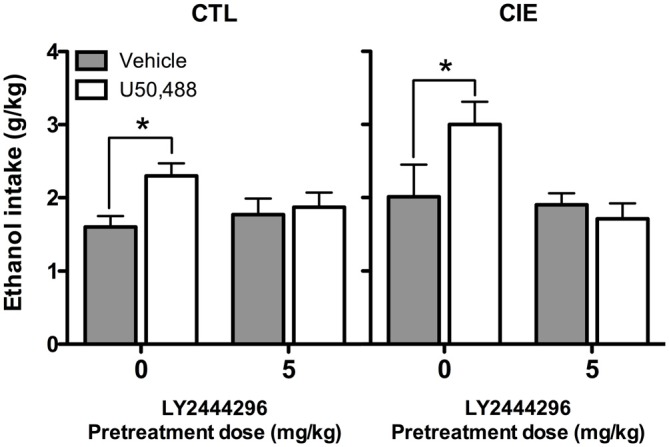
**Average ethanol consumption during final test week (g/kg).** For both CIE and CTL mice administered 5 mg/kg U50,488, pretreatment with the KOR antagonist LY2444296 blocked the increase in drinking observed in vehicle-treated mice (indicated by *).

Average weekly ethanol intake (g/kg) during Test Week 6 was analyzed using a 2 (Group) × 2 (LY2444296 dose) × 2 (U50,488 dose) factorial ANOVA. This analysis revealed significant main effects of LY2444296 Pretreatment [*F*_(1,71)_ = 5.90, *p* < 0.05] and U50,488 Treatment [*F*_(1,71)_ = 5.46, *p* < 0.05], as well as the interaction between these factors [*F*_(1,71)_ = 6.65, *p* < 0.05]. *Post hoc* analyses indicated that among vehicle-pretreated CIE and CTL mice, U50,488 (5 mg/kg) produced a significant increase in drinking. However, LY2444296 (5 mg/kg) pretreatment blocked this effect of the KOR agonist in both CIE and CTL groups of mice (Figure 6).

## Discussion

Results from the current series of experiments support a role for the dynorphin/KOR system in mediating stress-induced enhancement of ethanol drinking in mice with a history of dependence (CIE exposure). As we previously demonstrated (Lopez et al., 2016), daily exposure to FSS selectively increased ethanol consumption in CIE-exposed mice but did not alter drinking in nondependent (CTL) mice (Experiments 1 and 2). This stress effect was blocked by administration of the KOR antagonist LY2444296 (Experiment 2). Additionally, the effects of FSS were mimicked by administration of the KOR agonist U50,488. However, whereas FSS increased drinking only in CIE-exposed mice, pharmacological activation of KORs increased drinking in both CIE-exposed and CTL mice (Experiments 1 and 3). While the dynorphin/KOR system has been implicated in drinking in ethanol-dependent animals and stress-related behaviors, this study provides the first evidence that this system plays a role in the capacity of stressful experiences to selectively enhance drinking in dependent subjects.

### Forced Swim Stress Exposure Selectively Increases Ethanol Consumption in Mice with a History of Chronic Intermittent Ethanol Treatment

Results from the first and second experiments of the current study replicated our previous findings demonstrating that daily FSS exposure increases ethanol consumption in mice with a history of dependence (CIE exposure), but not in nondependent (air-exposed) controls (Lopez et al., 2016). A similar effect of FSS has been reported in rats given 24 h access to ethanol, with elevated consumption observed during and following FSS exposure in dependent (but not control) rats (Sommer et al., 2008). In the present study, FSS increased drinking in CIE-exposed mice on the same days that the stress exposure occurred, and this increase in ethanol consumption resulted in a nearly threefold increase in BECs. Of note, other studies have reported stress-induced increases in ethanol drinking in nondependent mice, but this effect was only observed when ethanol was made available for consumption after exposure to the stressor was terminated (e.g., Sillaber et al., 2002; Lowery et al., 2008; Norman et al., 2015; Hwa et al., 2016). Taken together, these results highlight the importance of the timing of stress exposure in relation to ethanol access, and also indicate that stress exposure (FSS in the present study) interacts with chronic ethanol exposure and withdrawal experience in a unique manner to promote elevated drinking in the context of dependence.

### KOR Blockade Prevents Stress-Induced Elevation of Drinking in Mice with a History of Chronic Intermittent Ethanol Treatment

Systemic administration of the KOR antagonist LY2444296 (5 mg/kg) prior to each drinking session blocked the FSS-induced increase in ethanol consumption observed in CIE-exposed mice. To our knowledge, this is the first evidence demonstrating that pharmacological antagonism of KORs prevents stress-induced increases in ethanol consumption. Previous studies have reported that other KOR antagonists reduce ethanol consumption in various models. For instance, the long-acting KOR antagonist nor-BNI was shown to block escalation of ethanol consumption induced by chronic vapor exposure in rats (Walker and Koob, 2008; Walker et al., 2011) and mice (Rose et al., 2015). JDTic was reported to attenuate ethanol-seeking behavior in nondependent Wistar and P-rats (Deehan et al., 2012; Schank et al., 2012), and another short-acting antagonist, LY2456302 was shown to reduce ethanol consumption in P rats (Rorick-Kehn et al., 2014). However, it is difficult to compare the effects of KOR blockade across studies, particularly since only one dose of the novel antagonist was tested in the current report. It will be important to test additional doses of LY2444296 in this model to determine whether higher doses reduce ethanol consumption in non-stressed subjects as well. While LY2444296 was effective in blocking stress-induced enhancement of drinking in CIE-exposed mice in the present study, the lack of an effect of this KOR antagonist on drinking in non-stressed CIE mice may be due to the 1 h drinking paradigm used that did not favor escalated intake. At present, it is unclear why some studies have demonstrated KOR antagonists to reduce ethanol consumption in nondependent animals (Deehan et al., 2012; Schank et al., 2012; Rorick-Kehn et al., 2014), while other studies indicate more selective attenuating effects for elevated drinking associated with dependence (Walker and Koob, 2008; Walker et al., 2011; Rose et al., 2015).

Because the KOR antagonist was administered after FSS exposure and prior to each ethanol drinking session in the present study, it is possible that the stress experience may have rendered mice more sensitive to the KOR manipulation. On the other hand, it may be that the KOR antagonist reduced stress-enhanced drinking in CIE mice by alleviating consequences of stress exposure (e.g., negative affect) that contribute to the elevation in drinking. Future studies that systematically examine the temporal relationship between stress experience, KOR antagonism, and ethanol access will be critical for addressing this issue.

### KOR Activation Increases Ethanol Consumption in Dependent and Nondependent Mice

Similar to the effects of FSS, systemic administration of the KOR agonist U50,488 significantly increased ethanol consumption in CIE-exposed mice; however, this effect was also observed in air-exposed control subjects. This finding was confirmed in a follow-up study (Experiment 3) that evaluated a wider range of U50,488 doses, but the increase in ethanol consumption did not vary as a function of dose. This was a surprising outcome given that the low doses of U50,488 evaluated in the present study have not previously been reported to influence drug reward (Schindler et al., 2010; Smith et al., 2012). Future studies will need to assess even lower doses of U50,488 to generate a dose-effect function and determine whether CIE and CTL mice exhibit differential sensitivity to the KOR agonist in this drinking model.

Our results are generally consistent with other studies that demonstrated similar behavioral effects of U50,488 and stress exposure on conditioned ethanol reward and consumption (Sperling et al., 2010), although it is unclear why only CIE-exposed mice were sensitive to FSS-induced increased drinking whereas both CIE and CTL mice were sensitive to the KOR agonist-induced increased drinking. Results from Experiments 1 and 3 also indicate that U50,488 produced a more robust elevation of ethanol consumption than FSS. It may be that direct pharmacological stimulation of KORs produces a general increase in ethanol consumption whereas FSS produces more complex effects than simply activating KORs. This, in turn, may explain why FSS interacts in a unique manner with CIE exposure to selectively increase drinking in dependent compared to nondependent mice. Thus, while systemic administration of a KOR antagonist completely blocked the ability of FSS to selectively enhance drinking in CIE-exposed mice, the mechanisms underlying this stress-CIE exposure interaction remain to be fully elucidated.

### Possible Mechanisms Underlying KOR Modulation of Stress-Ethanol Interactions

As noted above, the attenuation of stress-enhanced drinking in CIE mice by KOR antagonism may reflect one of several possible underlying mechanisms. We postulate that repeated cycles of CIE exposure combined with repeated FSS exposure activate the dynorphin/KOR system within brain stress circuitry, and that behavioral manifestations of this effect can be attenuated by blocking KORs. Indeed, chronic ethanol exposure has been reported to sensitize KORs in the nucleus accumbens core in monkeys and mice (Rose et al., 2015; Siciliano et al., 2015). Similarly, social isolation stress has been shown to sensitize these receptors in rats (Karkhanis et al., 2016). An alternative, but not mutually exclusive, explanation is based on the observation that stress exposure can enhance sensitivity to ethanol withdrawal symptoms (i.e., promoting escalated drinking via negative reinforcement). In this case, KOR blockade may prevent escalated drinking by attenuating ethanol withdrawal symptoms. Support for this idea comes from evidence indicating that KOR antagonists can attenuate withdrawal-related anxiety and other signs of negative affect (Schank et al., 2012; Valdez and Harshberger, 2012; Berger et al., 2013), without altering physical symptoms of dependence (Kissler and Walker, 2015).

Increased activity of the dynorphin/KOR system within the amygdala has been suggested to play a significant role in mediating the negative emotional aspects of chronic ethanol exposure and withdrawal (e.g., Valdez and Harshberger, 2012; Berger et al., 2013). Thus, the amygdala appears to be a primary brain region for KOR modulation of stress interactions with the motivational effects of ethanol. A study that assessed c-Fos activation following footshock stress in wild-type and prodynorphin knockout mice with or without a long history of ethanol consumption implicated the basolateral amygdala (BLA) as a potential locus of dynorphin-mediated interactions of stress and ethanol (Rácz et al., 2013). Among wild-type mice in this study, a history of chronic ethanol consumption resulted in greater stress-induced c-Fos activation in the BLA relative to their ethanol-naïve counterparts. Conversely, among prodynorphin knockout mice, stress-induced c-Fos activation was blunted in ethanol-exposed mice compared to ethanol-naïve subjects (Rácz et al., 2013). The CeA also appears to be an important brain region where KORs exert effects on ethanol-related signaling (Kang-Park et al., 2013; Gilpin et al., 2014; Kissler et al., 2014). Site-specific administration of the KOR antagonist nor-BNI has been shown to reduce chronic ethanol-induced escalated ethanol consumption.

Interestingly, increased activity of another stress-related peptide system, CRF, in the CeA has been implicated in stress and motivational effects of ethanol associated with dependence (Funk et al., 2006; Finn et al., 2007; Heilig and Koob, 2007; Becker, 2012). Further, recent work has suggested interactions between KOR and CRF systems in the CeA that influence ethanol’s effects on GABA-ergic transmission (Kang-Park et al., 2015). As numerous studies have demonstrated that interactions of dynorphin and CRF can influence stress, anxiety, and drug-seeking behavior (e.g., Valdez et al., 2007; Land et al., 2008; Bruchas et al., 2009; Funk et al., 2014), the potential interaction between these peptide systems in mediating stress-enhancement of drinking in the context of dependence should be addressed in future studies. Further, evidence that KORs interact with noradrenergic and serotonergic signaling to regulate the influence of stress on drug reward and reinstatement (Land et al., 2009; Al-Hasani et al., 2013) suggests that the effects of KOR manipulation on stress-induced ethanol consumption may reflect integrated effects mediated through multiple neurotransmitter systems. Additional research will be required to elucidate the precise mechanisms by which KOR blockade abolishes stress-induced drinking in subjects with a history of chronic ethanol exposure.

### Therapeutic Potential of KOR Antagonists for Treatment of Alcohol Use Disorder

Results of the current study provide evidence that pharmacological manipulation of KOR activity can modulate stress-induced increases in ethanol consumption in mice with a history of CIE exposure. As such, these results suggest that KORs may be a promising target for therapeutic intervention of alcoholism, particularly among individuals that engage in harmful levels of drinking that is principally driven by stressful experiences.

## Author Contributions

RIA, MFL, and HCB conceived and designed the study. RIA conducted the experiments, analyzed all data, and wrote the manuscript. HCB revised the manuscript, which all authors read and approved.

## Conflict of Interest Statement

The authors declare that the research was conducted in the absence of any commercial or financial relationships that could be construed as a potential conflict of interest.
